# *Meloidogyne graminicola*—A Threat to Rice Production: Review Update on Distribution, Biology, Identification, and Management

**DOI:** 10.3390/biology10111163

**Published:** 2021-11-11

**Authors:** Leidy Rusinque, Carla Maleita, Isabel Abrantes, Juan E. Palomares-Rius, Maria L. Inácio

**Affiliations:** 1 Instituto Nacional de Investigação Agrária e Veterinária (INIAV, I.P.), 2780-159 Oeiras, Portugal; leidy.rusinque@iniav.pt (L.R.); lurdes.inacio@iniav.pt (M.L.I.); 2 Centre for Functional Ecology—Science for People & the Planet, Department of Life Sciences, University of Coimbra, Calçada Martim de Freitas, 3000-456 Coimbra, Portugal; isabel.abrantes@uc.pt; 3 Chemical Process Engineering and Forest Products Research Centre, Department of Chemical Engineering, University of Coimbra, Rua Sílvio Lima Pólo II—Pinhal de Marrocos, 3030-790 Coimbra, Portugal; 4 Institute for Sustainable Agriculture (IAS), Spanish National Research Council (CSIC), Avenida Menéndez Pidal s/n, 14004 Córdoba, Spain; palomaresje@ias.csic.es; 5 GREEN-IT Bioresources for Sustainability, ITQB NOVA, Av. da República, 2780-157 Oeiras, Portugal

**Keywords:** damage, hosts, life cycle, plant-parasitic nematode, rice root-knot nematode

## Abstract

**Simple Summary:**

New risks to plant health are constantly emerging. Such is the case of the rice root knot nematode *Meloidogyne graminicola*, adapted to flooded conditions and representing a risk to all types of rice agro-systems. It has been recently detected in Italy and added to the European and Mediterranean Plant Protection Organization (EPPO) Alert List. The presence of this nematode in Europe poses a threat to rice production, as there is a high probability to spread, due to trade activities and climate changes. In view of its importance, an extensive updated review was carried out.

**Abstract:**

Rice (*Oryza sativa* L.) is one of the main cultivated crops worldwide and represents a staple food for more than half of the world population. Root-knot nematodes (RKNs), *Meloidogyne* spp., and particularly *M. graminicola*, are serious pests of rice, being, probably, the most economically important plant-parasitic nematode in this crop. *M. graminicola* is an obligate sedentary endoparasite adapted to flooded conditions. Until recently, *M. graminicola* was present mainly in irrigated rice fields in Asia, parts of the Americas, and South Africa. However, in July 2016, it was found in northern Italy in the Piedmont region and in May 2018 in the Lombardy region in the province of Pavia. Following the first detection in the EPPO region, this pest was included in the EPPO Alert List as its wide host range and ability to survive during long periods in environments with low oxygen content, represent a threat for rice production in the European Union. Considering the impact of this nematode on agriculture, a literature review focusing on *M. graminicola* distribution, biology, identification, and management was conducted.

## 1. Introduction

Rice (*Oryza sativa* L.) is the third most important cereal crop in the world, just behind wheat and maize, playing a strategic role in solving food security issues. New risks to plant health are constantly emerging. Many nematodes in rice have been detected and described, but only a few have harmful effects on rice production, such is the case of the rice root-knot nematode (RKN) *Meloidogyne graminicola* Golden and Birchfield, 1965 (*Mg*) [1], recently detected in Italy and added to the European and Mediterranean Plant Protection Organization (EPPO) Alert List [2]. *Mg* is considered a major threat to rice production, particularly in Asia. Projections by the Intergovernmental Panel for Climate Change indicate that there will be an increase in mean annual temperature and rainfall in South Asia, West Africa, and Europe. The elevated temperature and moisture may result in an increasing rate of infection, development, and reproduction, causing shifts in *Mg* abundance and geographic distribution. Such effects may have a detrimental impact on rice in temperate regions. Furthermore, *Mg* is a clear example of how alterations in rice production (shortage of water due to socioeconomic pressure and climate change) contributed to changes in its status as the major plant-parasitic nematode (PPN) in rice. An effort has been made to gather all the information regarding several aspects of *Mg* to present it as a comprehensive review on rice RKN. 

## 2. *Meloidogyne graminicola*—Origin and Distribution

The rice RKN, *Mg*, was first isolated in India by Israel et al. [3], but it was only described in 1965 when it was found on the roots of barnyard grass (*Echinochloa colonum*) in Baton Rouge, Louisiana, USA [4]. Since then, this nematode has been reported from the USA on rice and weeds in Louisiana, on grass in Georgia and Mississippi, and on sandbur (*Cenchrus* spp.) in Florida [5,6,7,8]. Its occurrence has been widely accounted in rice fields in several Asian countries [9,10,11] and also in South Africa, Colombia, Brazil, and Italy [12,13,14]. 

*Mg* has been reported to parasitize primarily in irrigated and rainfed rice in South and Southeast Asian countries, such as China, India, the Philippines, Burma (Myanmar), Bangladesh, Pakistan, Laos, Thailand, Vietnam, and Nepal [15,16,17]. In China, it was first found on *Allium tistulosum* in the Hainan province by Zhao et al. [18]. More than a decade later, it was detected associated with rice and other hosts including weeds in the provinces of Anhui, Fujian, Hainan, Hunan, Hubei, Zhejiang, Jiangxi, and Sichuan, causing a severe incidence in the Hunan province [19,20,21,22].

In India, this nematode was first isolated in the county of Orissa from upland rice soils by Israel et al. [3]. Since then, it has been found infecting rice in the provinces of Andaman and Nicobar Islands, Assam, Andhra Pradesh, Bihar, Gujarat, Himachal Pradesh, Jammu and Kashmi, Karnataka, Kerala, Madhya Pradesh, Manipur, Orissa, Tamil Nadu, Tripura, and West Bengal [23,24]. In 1971, its presence was referred in Thailand, causing typical root galls in entire rice-growing areas and in nursery seedbeds [25], and in Bangladesh, where it has been often associated with deepwater and pre-monsoon upland rice systems [26,27,28]. Minor infestations were reported in lowland rainfed rice areas [28]. Nonetheless, in the northwest of Bangladesh, where the dominant cropping system is lowland rainfed alternated with wheat, severe infestations of *Mg* were observed [29].

Later, in the 1990s, *Mg* was reported infesting rice fields in Sri Lanka, where it is now dispersed into major rice-growing areas of the country [30,31,32]. In a study performed in Vietnam, in 1992, to determine the PPN in deepwater rice systems, *Mg* was identified for the first time as one of the main causes of high yield losses of rice [33]. In Pakistan, during a survey in rice fields of Sheikhupura (Punjab), Munir and Bridge [34] reported its presence for the first time in the country and in 2007, *Mg* was detected in Nepal [35].

The occurrence of *Mg* in Africa was recorded on grass roots of *Paspalum* sp. in the South East region of Antsirabe, and its identification was based on morphological traits [36]. Later, in 2014, during a survey carried out in 14 sites distributed along a NW/SE axis between the towns of Marovovay and Manakara, *Mg* was found [37].

The first report of *Mg* in South America was by Monteiro et al. [38] in cyperaceas collected in Presidente Prudente, São Paulo, Brazil. However, only in 1991, Sperandio and Monteiro [39] first reported and described the species in the municipality of Palmares do Sul (Rio Grande do Sul) and, in 1994, Sperandio and Amaral [40] found *Mg* in other municipalities in the south of Rio Grande do Sul. The latest reports confirm the presence of the rice RKN in the region [41,42].

In Ecuador, *Mg* was first identified in 1987, in the “Sausalito” village located in the corner of Puerto Inca, province of Guayas, in a field planted with the cultivar Oryzica 1. In surveys conducted in the Provinces of Manabí, Guayas, and Los Ríos, *Mg* was not found in any other field planted with rice. Nevertheless, by 2000, it had already been disseminated to all rice fields of the Province of Guayas and, in 2002, it was present in the Province of Los Ríos [43]. In a new survey conducted in 2015 in the provinces of Guayas and Los Ríos, the rice RKN was found to be the most widespread, occurring in both rainfed lowland and irrigated areas in high densities [13]. 

In Colombia, Goméz et al. [44] reported the presence of galls in the roots of rice plants in the county of Tolima, Ibague. Thirteen years later, in a survey programme established by the Colombian rice federation “FEDEARROZ”, Bastidas and Montealegre [45] described the symptoms of a new rice disease denominated as “Entorchamiento” and concluded that it was caused by nematodes of the *Meloidogyne* genus. The species *Mg* was later identified, on the basis of morphological and biometrical characters, in other counties and its presence confirmed in other rice production zones, corroborating its spread throughout the country [46,47].

In Europe, *Mg* was detected, in July 2016, in several rice fields of northern Italy in the Piedmont region, being the first report of its presence in the EPPO region [14]. Due to this detection, the EPPO decided to include *Mg* in the Alert List A2 in 2017. Following the first report, it was detected in the Lombardy region, province of Pavia [2].

**Figure 1:**
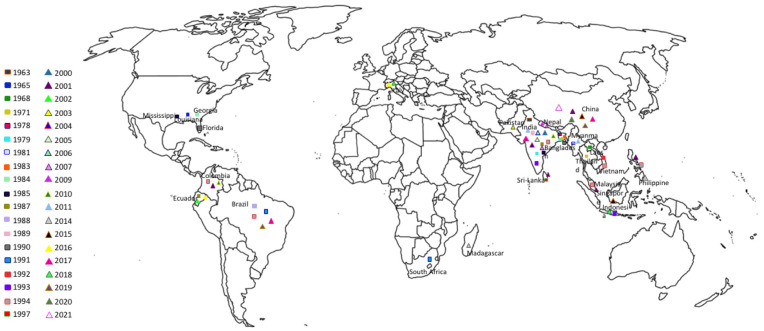
Geographical distribution of *Meloidogyne graminicola*.

This *Meloidogyne* species is present almost in every continent (Table 1. Figure 1). Such occurrence and increase detection draws attention to its potential to affect temperate rice agro-systems adversely.

## 3. Life Cycle and Symptoms

*Mg* is a facultative meiotic parthenogenetic species with the probability of occurring amphimixis being very low [79,80]. The infective second-stage juvenile (J2) move through the soil to find a suitable host and penetrate the root near the tip. They migrate intercellularly towards the region of cell differentiation, close to the root meristem, inducing a permanent feeding site in the stele [81,82]. Once established in the roots, J2 become sedentary and flask-shaped and undergoes three molts to become third (J3) and fourth stages (J4) and adult stage. Hyperplasy and hypertrophy of surrounding cells cause the formation of macroscopically visible galls on the root system [1,83,84]. These galls with a characteristic hook shape are located mostly at the root tips, affecting root development and physiology, and a profuse proliferation of very slender and fluffy roots that lead to substantial yield losses [12,85,86]. Females remain within the galled roots, and eggs are deposited in a gelatinous matrix (egg mass) inside the root cortex. The first-stage juveniles (J1) develop inside the egg and molt to become J2. After hatching, the J2 can be released into the soil or remain within the gall to migrate and establish new feeding sites, inducing the formation of new galls [27,87,88,89]. This unusual way of laying eggs is an advantage as it allows *Mg* to complete its life cycle without leaving the host. Up to 50 egg-laying females can be found in a single gall, indicating that infection can be extremely high [12]. As *Mg* is unable to penetrate rice roots in flooded soils, it has been reported that under continuously flooded conditions, egg masses remain viable for as long as 14 months and J2 for at least five months, resuming their activity by attacking the root tips when fields are drained [27,90].

The most common underground symptom is the characteristic hook shape of the galls, as referred before. Additionally to the consumption of cytoplasmic content of giant cells by the nematode, the galling produced by *Mg* provokes an alteration of the root vascular system by disrupting water and nutrient transport from the roots to the aboveground parts, resulting in loss of plant vigor, poor growth, and yield reduction [91]. To maintain a compatible host–parasite relationship, *Mg* meddles and manipulates the defense mechanism of the plant, making it unable to prevent the nematode penetration and development [80]. Infestations of *Mg* cause a reduction in phenols and changes in plant immunity gene expression in the shoots and roots, causing greater susceptibility to the rice blast pathogen, *Pyricularia oryzae*, and fungus from soil, such as *Fusarium moniliforme* [3,92,93].

Aboveground symptoms due to *Mg* infection include patches in rice fields, stunted appearance, chlorotic leaves, early flowering and maturation, and few chaffy grains on the panicles on heavily affected root systems [80,94,95]. These symptoms are similar to that attributed to nutritional and water-associated disorders or to secondary diseases. The degree of symptom manifestation differs with time of infection, age of the plants, and climatic conditions [17]. A reduction in chlorophyll content and changes in photosynthetic rates were also reported by Swain and Prasad [96,97]. Losses in flooded rice fields occur when infected seedlings fail to develop, leaving patches of open water in the fields [27]. Overall, symptoms observed in infested upland and lowland rice fields from different geographical locations reported by several researchers match among them. For instance, in Italy, the fields showed patches, with plants exhibiting poor growth and stunting and roots having galls of different shapes and sizes [14]. In India, surveys carried out in rice fields, from different districts, a loss of vigor, reduced tillering, poor growth, and galls were detected [24,98,99].

Khan et al. [100] observed that in some species of weeds, the egg masses were found within the galls, while others had small galls with egg masses on the root surface or heavy root galling and large egg masses. In Bangladesh, *Mg* was associated with yellowing and stunting of deep-water rice and drowning of plants when they remain submerged and die after rapid and deep flooding [50,101]. In China, the symptoms included chlorotic leaves on heavily affected root systems, while root tips become swollen and hooked [102,103]. In South America, newly emerged leaves appear distorted and crinkled along the margins and roots show the characteristic hook-like galls [41,42,46,104].

*Mg* reproduces relatively fast on rice, depending on temperature and climatic conditions, when compared with other RKN species. Several authors reported that the *Mg* life cycle varies considerably, ranging from a very short life cycle of only 15 days at 27–37 °C [105,106] to a rather long life cycle of up to 51 days in some regions of India [107,108]. On average, *Mg* can complete its life cycle within 19 to 27 days during the early summer, but the period can extend by 5 to 12 days [27,105,108,109,110]. For instance, isolates from Bangladesh had a very short life cycle on rice of <19 days at temperatures of 22–29 °C [27] and an isolate from the USA completed its life cycle in 23–27 days at 26 °C [105]. Due to the short life cycle, the presence of even a small number of *Mg* J2 at planting can lead to an increase of the population density during a single crop cycle [111].

## 4. Damage/Crop Losses in Rice

*Mg* is the most prevalent PPN on rice and considered a major threat to rice as yield losses can reach up to 70% [12,94,112]. *Mg* densities of 120, 250, and 600 eggs/plant in seedlings 10, 30, and 60 days after planting were reported by Rao et al. [110], causing 10% losses. In a later study, Cuc and Prot [78] stated that a density of 100 J2/g root could be considered as high infestation. Most recently, Win et al. [74] found that population densities could exceed 1000 J2/g root with 12–16 galls/plant, contributing to a 65% yield reduction. It has also been found that there is a decline in yield when more than 75% of the roots are affected by nematodes [32]. Additionally, the water regime is an important environmental factor that influences the development and population dynamics of *Mg*, and the damage and yield loss that it can cause to rice. Soriano et al. [91] showed that rice cultivar tolerance to *Mg* varies with the water regime and that yield losses may be prevented or minimized when the rice crop is flooded early and maintain inundated until harvesting. For example, losses in lowland rainfed rice in Bangladesh can range between 16 and 20%, while in India, losses range between 16 and 32% under irrigated conditions and between 11 and 73% under flooded conditions [102,113]. In China, the highest incidence of the disease is in the Hunan provinces, exceeding 85% in infested paddy fields [19]. Furthermore, reports of *Mg* infestations in rice–wheat agroecosystem of India, Nepal, and Pakistan suggest that the damage caused by the rice RKN may be responsible for the poor productivity in this cropping system [10,11,35,114].

Changes in agricultural policy and adoption of new rice production technologies in South East Asian countries have influenced the status of the rice RKN problem [75]. For instance, in the Philippines, *Mg* became a major constrain due to the intensification of rice cropping and shortage of water supply. This situation forced the farmers to grow direct wet seeding, and intermittent irrigation, providing favorable conditions for *Mg* infestation and increasing the economic losses [9,75]. In India, the system of rice cultivation shifted to the so-called “system of rice intensification practice”, where a new ecological condition is being developed through modification of rice cultivation practices that includes planting younger and tender seedlings, the creation of greater aeration in soil, and regulation in irrigation. All these conditions provide a suitable environment to increase the infestation levels of the rice RKN [112,114,115].

Spatio-temporal studies have also demonstrated that densities of *Mg* J2 in the soil fluctuate throughout the year [116]. Moreover, *Mg*’s ability to survive and reproduce in off-seasons on weeds and forage crops contributes to increase the population levels in the soil, and rice infection in the next season [35]. Besides alternative hosts and irrigation, the soil type influenced the tolerance of plants to *Mg* and showed differences in the multiplication of the nematode [91]. Studies have also revealed that infestation levels depend on the rice cultivar [117,118], and the aggressiveness differs between populations, suggesting intraspecific variability [35,119]. It was also found that *Mg* consists of more than one race. In fact, populations from Florida have shown less aggressiveness and difference on the host infection and reproduction patterns than the Asian populations, and populations from Vietnam are not able to reproduce on tomato (*Solanum lycopersicum*), soy (*Glycyne max*), or green beans (*Phaseolus vulgaris*), despite these species being reported as a host of *Mg* [16,119,120].

## 5. Host Plants

In addition to the main host, rice, *Mg* has a wide range of alternative hosts, including cereals and grasses, as well as dicotyledonous plants [15,120,121] (Table 2). Forty-six weeds commonly growing in or around rice fields were assessed for host suitability and were found to be moderate to good hosts of *Mg* [122]. Khan et al. [100] reported 17 weed species and, in 2009, Rich et al. [15} reported 24, which supported the survival and multiplication of *Mg* in the field, acting as a reservoir of nematodes when rice is not present during crop rotations [15] (Table 3). Furthermore, it was believed that *Mg* caused yield losses only in rice; however, a reduction of the root length of onion (*Allium cepa*) was observed, with yield losses of 16–35% in the Philippines [76]. In Nepal, India, Pakistan, and Bangladesh, it is considered a threat to wheat crops and to vegetables, such as aubergine (*S. melongena)*, tomato, and okra (*Abelmoschus esculentus*) [10,122,123,124,125].

## 6. Identification Approaches: From Classical to Molecular Methods

The identification of *Mg* is complex and crucial to understand the host–parasite relationships and to implement appropriate management strategies. Similar to the identification of other *Meloidogyne* species, the classical methods are based on the symptoms (root galls), morphology, biometrics, and differential host range tests [139,140,141,142,143]. The *Meloidogyne* ‘graminis-group’, the most defined group within the genus, with some species being morphologically extremely similar, including *M. graminicola, M. graminis*, *M. hainanensis*, *M. lini*, *M. oryzae*, *M. salasi*, and *M. triticoryzae* [48,144]. In studies performed by Pokharel et al. [16] and Luo et al. [103], morphometrics among and within populations did not correlate with the geographic origin. Pokharel et al. [35] mentioned that J2 from Bangladesh and the United States were significantly longer and smaller than the Nepalese, and presented minor variability among them. These morphometrical differences might be due to different geographical origin and intraspecific variability, or phenotypic plasticity commonly exhibited by nematodes [16,69,145]. Morphological features, such as the female’s perineal patterns, female excretory pore position in comparison to stylet length, the position of hemizonid and tail shape in J2, as well as body, stylet, and tail measurements, are considered valuable tools for *Mg* identification due to their low cost, but they need specialized technicians to identify and measure these characters.

Other identification methods include enzymatic studies [146]. Isozyme phenotyping has demonstrated that the major species of *Meloidogyne* (*M. incognita, M. javanica, M. arenaria*, and *M. hapla*) can be differentiated by species-specific enzyme phenotypes, esterases (EST), malate dehydrogenase (MDH), superoxide dismutase (SOD), and glutamate-oxaloacetate transaminase (GOT), which can be revealed by polyacrylamide gel electrophoresis (PAGE) and a specific staining technique [147]. Esterase activity has demonstrated to be highly polymorphic and the most useful in the identification of the species. Furthermore, progresses in electrophoretic procedures have made possible and practical the detection of different EST phenotypes of a single female [148]. The main drawback of this method is that it requires adult females at a specific developmental stage for accurate diagnosis, which hinders its use in routine examination of soil samples that often contain only J2 or males.

Esbenshade and Triantaphyllou [146] described, in 1985, in one population of *M. oryzae*, an esterase phenotype designated as VS1 (very slow with one band), as having a large drawn-out band of high enzymatic activity. The same phenotype with a slightly slower band (Est VS1) was also detected in a population of *Mg* and two undescribed populations isolated from rice, which were later described as *M. salasi*. Since the VS1 phenotype did not characterize a single species, it remained the EST phenotype of these species. This fact shows the inaccuracy of this technique when identifying closely related species with similar phenotypes, such as *M. salasi, M. graminicola,* and *M. graminis*. Populations of *M. oryzae* showed a pattern O1 in an integrative taxonomy study performed by Mattos et al. [149]. Other studies have shown a high variability on *Mg* populations [48,149,150], which poses a risk of misidentification. Moreover, MDH enzymatic phenotype N1 is shared among *Meloidogyne* species, i.e., *M. chitwoodi* and *M. salasi* [48,146].

In order to assist *Mg* identification, the application of molecular methods has been used with partial success; in particular, sequences of nuclear ribosomal (rDNA) and mitochondrial DNA (mtDNA) as molecular markers for sequence comparison [16,35,151,152]. In 2017, Salalia et al. [69] and Fanelli et al. [14] found high variability within isolates of *Mg* from India and Italy, the USA, and China and, based on cytochrome oxidase subunit II and 16S ribosomal RNA (COXII-16S rRNA) genetic analysis, considered the existence of two groups of *Mg*: group A, which clusters the populations from the USA and Italy, and group B with those from China. According to Pokharel et al. [35], the analysis of internal transcribed spacer (ITS) sequences as genetic markers allowed the detection of two groups in *Mg* Nepalese populations: group I, clustering with *M. trifoliophila*, and group II with *Mg* from the USA. A new race of *Mg* from Florida, USA, which did not parasitize rice was also identified by Pokharel et al. [16], based on the ITS region and morphological and morphometric characters that are not species specific. Furthermore, Bellafiore et al. [119] and Salalia et al. [69] detected great morphological variability among populations of *Mg* from India and Vietnam, and using an ITS marker, concluded that all the isolates belonged to *Mg*. Salalia et al. [69] even suggested the presence of cryptic species among Indian populations. On the other hand, Htay et al. [152], when analyzing ITS-rDNA sequences, from the same individual or from different nematodes from the same sample noted that there was nucleotide variability. These differences could be attributed to variations among copies of the ITS within an individual, or to errors arising through PCR amplification, cloning, or sequencing [35].

Several molecular methods have been developed to detect *Mg*: (1) ITS-PCR-RFLP [14]; and (2) diagnostic SCAR marker [119,149,152] for rapid and reproducible identification of *Mg*. However, Negretti et al. [48] and Soares et al. [150] showed inespecificity associated with *M. oryzae* and *M. ottersoni*; (3) real-time PCR primers for the quantification of *Mg* in soil [153,154], with the drawback that some primers amplifying DNA of the closest non-target species (*M. incognita* and *M. hapla*) or not widely tested against other species; and (4) mediated isothermal amplification [154].

## 7. Genomic and Transcriptomics

The mitochondrial genome of three *Mg* isolates from the Philippines, China, and India has been sequenced [155,156,157]. Somvanshi et al. [157] included the first genome draft from India, but, recently, Phan et al. [158] generated a highly contiguous reference genome (283 scaffolds with an N50 length of 294 kb, totaling 41.5 Mb), with the highest completeness scores currently published for *Meloidogyne* genomes. This genome assembly constitutes a great improvement and represents a valuable molecular resource for future phylogenomic studies and evolutionary history reconstruction. Somvanshi et al. [159] improved the genome assembly of the Indian isolate IARI using long-read sequencing. Comparison of both genomes displayed a high correlation between them, 35.9 Mb of 36.86 Mb assembly in the IARI isolate anchored onto the 41.5 Mb of the Mg VN18 assembly [159]. However, there are important differences in the protein-coding genes between both genome assemblies (14,602 (IARI) vs. 10,284 (VN18)), suggesting that the different sequencing platforms used in both assemblies have captured unique features of the *Mg* genome.

Genomic tools have been developed to help understand the molecular responses of plants to nematode infection. Therefore, transcriptome analyses have become a useful tool to profile the expression of several key genes throughout the infection process in the feeding site, and systemically in the plant and nematode [82]. Previous research evidenced that plant–nematode interactions affect the expression of genes associated with plant immune response [80,89]. Differential expression of plant defense genes and other related changes in host plants are mainly modulated by phytohormones, such as salicylic acid, jasmonic acid, and ethylene. Research demonstrated that RKN represses the jasmonic acid pathway and a few phenylpropanoid pathway genes during the establishment in the rice plants [160,161,162].

PPN can secrete effector T-proteins into the host tissue to facilitate their infection by reprograming the host metabolism, or by preventing the plant defense responses. These effectors also have a role on nematode migration inside the plant roots and are required to initiate and maintain the feeding sites [163,164]. Haegeman et al. [165] and Petitot et al. [166] analyzed the transcriptome of *Mg* J2 to identify genes and its pattern of expression during infection of rice plants, leading to the identification of new candidate effector genes: *Mg*40980 gene encoding a metallothionein; *Mg*12322 and *Mg*28330, encoding Cys-rich proteins; and *Mg*11937 gene, encoding a venom allergen-like protein, among others. Over the past years, novel *Mg* effectors playing a role in nematode parasitism were functionally characterized, including pioneer genes [167,168], a C-type lectin [169], and a protein disulfide isomerase [170]. In 2020, Petitot et al. [171] analyzed mRNA-seq data derived from nematode-infected rice tissue to identify nematode transcripts specifically expressed when the nematode resides inside the plant, through a comprehensive transcriptome analyses of J2 and rice infected tissues until the development of young adult females. Dash et al. [172] delivered a transcriptome comparison of nematode-resistant and -susceptible rice plants in the same genetic background. Through RNA-seq, the molecular mechanisms that confer resistance to *Mg* during early infection were identified. These findings provide a global view of the genes expressed in the rice–*Mg* interaction, highlighting that *Mg* adapts its gene expression depending on the plant genotype. It may also suggest that the initial resistance to nematode infection is mediated by nematode recognition followed by the expression of plant defense genes and secondary metabolites.

Nevertheless, additional efforts are required to identify the underlying pathways and mechanisms responsible for the resistance of rice to *Mg*, as well as important genes for successful infection of the plant by *Mg*.

## 8. Management

The best strategy for management of *Mg* is to prevent the movement of plant and soil that in some cases may adhere to machinery or tools. In a recent pest risk analysis for *Mg* in Italy, it was concluded that the main ways of dispersion of this nematode are likely to be through the movement of infected plants and infested soil, non-host plants that may have grown near areas infested with *Mg*, and floating roots or plant material in the water [121]. Migrant waterbirds, machinery, and travelers were considered a secondary source of entrance. On the other hand, changes in the water regime (intermittent irrigation or water shortages) in many parts of the world are also contributing to the spread and infectivity of the nematode.

To minimize the losses resulting from *Mg*, management strategies are of extreme importance, and studies have shown that a combination of methods is the best approach to control this nematode in rice fields. The methods that have been applied to control *Mg* include the use of synthetic nematicides, known as the most efficient strategy, cultural methods, biological agents, and natural nematicides.

Some synthetic nematicides were, recently, strictly regulated or banned from the market, due to the adverse impacts on the environment and human health, reducing the alternatives for RKN control. Cultural methods (fallowing, soil solarization, crop diversification and rotation, etc.) also appeared to have some efficacy. For instance, crop rotation studies with non-host crops, like sweet potato (*Ipomoea batatas*), cowpea (*Vigna unguiculata*), sesame (*Sesamum indicum*), castor (*Ricinus communis*), sunflower (*Helianthus annuus*), soybean (*Glycine max*), turnip (*Brassica rapa* subsp. *rapa*), and cauliflower (*Brassica oleracea* var. *botrytis*), showed to prevent *Mg* development [110,132,173]. Nonetheless, none of these practices have gained importance among farmers, because of the high cost and unsatisfactory results. Furthermore, as many weeds found in rice fields are hosts for *Mg*, serving as nematode reservoirs for the next crops, a weed management programme must be implemented to maintain a low nematode population in infested fields.

Alternative strategies, such as the “rice field flooding technique”, used by the Italian National Plant Protection Organization (Ministerial Decree of 6 July 2017) to control *Mg*, had some effect on the nematode population densities. *Mg* can still propagate under flooding conditions, but the damage induced by this nematode is lower than in shallow intermittently flooded fields [80,174]. Nevertheless, this method of control also has some limitations, as there are areas where this practice is not applicable due to the soil structure, characterized by a low water retention capacity, or restriction in water use. Another approach explored by Sacchi et al. [174] was the use of rice plants as trap crops. Preliminary results indicate that trap cropping for the management of the rice RKN is efficient in most rice-growing areas, especially those with water shortages. However, additional studies are required to establish the most effective number of trap crop cycles that are necessary to reduce *Mg* population density. Additionally, this technique, in our opinion, could be highly influenced by climate in northern latitudes in order to sow rice in advance and the cost of machinery and water.

The use of biological control agents, such as the fungi *Paecilomyces lilacinus*, *Trichoderma harzianum, T. viride*, and other *Trichoderma* spp.; the bacteria *Bacillus subtilis*; and the rhizobacterium *Pseudomonas fluorescence*, have shown promising results against *Mg* [175,176,177,178]. Studies by Amarasinghe and Hemachandra [178], in Sri Lanka, revealed that *T. viride* reduces gall formation and production of egg masses, which represents a potential strategy to be included in integrated pest management programs.

Similarly, the use of essential oils (EOs) has been explored to control RKN, as an alternative to the synthetic nematicides. The nematicidal effects of EO from spices and medicinal plants on RKN have been widely reported. The high effect of *Cymbopogon* spp. EO (*C. martini motia*, *C. flexuosusand*, and *C. winterianus*) on J2 mortality has been described [179,180,181]. Chavan et al. [182] stated that basil (*Ocimum basilicum*), peppermint (*Mentha×piperita*), and lemongrass (*Cymbopogon citratus*) EOs have nematicidal properties against *Mg*. In order to confirm the efficacy of these EOs, the in vitro tests must be complemented by in vivo soil-based experiments.

Host plant resistance is an environmentally friendly and cost-effective strategy to mitigate damage caused by *Mg*. A promising alternative for the control of *Mg* is the screening of germplasm for genotypes that are resistant/tolerant and the development of resistant/tolerant cultivars [80,108,183]. Resistance sources against *Mg* have been identified in African wild accessions of rice (*O. glaberrima* and *O. longistaminata* and *O. rufipogon*) [184], and variability to a certain extent has been perceived [162]. Wild accessions that are partially or fully resistant to *Mg* can therefore act as resistant donors for interspecific crosses with Asian cultivars of rice [184,185]. Introgression of *O. glaberrima* into *O. sativa* has led, for example, to the new rice for Africa, NERICA cultivars [186], but the introgression has not been very successful [187]. Therefore, natural resistance in *O. sativa* cultivars is potentially very important. In Asian rice, using the Bala and Azucena mapping population, chromosomes 1, 2, 6, 7, 9, and 11 have been reported as having quantitative trait loci (QTL) for partial resistance to *Mg* [111]. Mapping of *Mg* resistance on chromosome 10 in Asian rice (cv. Abhishek), using bulk segregant analysis, was reported by Mhatre et al. [188]. A hypersensitivity-like reaction to *Mg* infection found in the Asian rice cv. Zhonghua 11 suggests that resistance to *Mg* was qualitative rather than quantitative, involving (a) major gene(s) [189]. Galeng-Lawilao et al. [190] reported the main effect QTL for field resistance in Asian rice on chromosomes 4, 7, and 9 plus two epistatic interactions (between loci on chromosome 3 and 11, and between 4 and 8).

Few studies have used genome-wide association studies (GWASs) as a viable strategy to identify novel QTLs for PPN resistance or susceptibility in different plants [191,192]. For example, Dimkpa et al. [191] confirmed the robustness of GWAS to screen for rice–nematode interactions and identified two resistant accessions (Khao Pahk Maw and LD 24). Studies carried out, in India, by Hada et al. [193] allowed the identification of 40 highly resistant accessions. Alternatively, the profiling of the defense response of 36 rice cultivars to *Mg* infection revealed a variation in the expression of plant defense genes [194]. Among all the selected plant defense genes, the expression of mitogen-activated protein kinases (MAPK20), isochorismate synthase genes (ICS1), nonexpressor of pathogenicity expression genes1 (NPR1), phytoalexin-deficient 4 (PAD4), allene oxidase synthase (AOS2), jasmonic acid-inducible rice myb gene (JAMYB), and 1-aminocyclopropane-1-carboxylic acid oxidase (ACO7) was upregulated, possibly providing resistance against *Mg.* This observation matches the insignificant expression in the susceptible genotypes. These outcomes are significant and can be exploited for breeding purposes.

## 9. Conclusions

Climate changes and the trade activity are supporting the northerly movement of pests, which means temperate agro-systems are likely to be affected. Higher temperatures and moisture may result in an increasing rate of infection, development, and reproduction, causing shifts in abundance and geographic distribution. Such is the case of *Mg* that has recently been detected in Italy, posing a threat to EU rice production and other economically important crops. Its adaptability to flooded conditions means that *Mg* can be found in both upland (rainfed) and lowland (irrigated) rice, and in deep-water ecosystems. This rice RKN is capable of completing several generations within a single growing rice season, promoting the rapid build-up of damaging population densities and infection of more than 150 plants. Besides, there are no effective and sustainable management strategies available. Therefore, future research should be focused on the *Mg* distribution, biology, and on new approaches for the identification and management of this RKN species, which can be considered a threat to rice production.

## Figures and Tables

**Table 1:** Distribution of *Meloidogyne graminicola* in Africa, America, Asia, and Europe.

Distribution	Year	References
**Africa**		
Madagascar	2014	[37]
South Africa	1991	[36]
America (North-USA)		
Florida	2003	[8]
Georgia	1984	[6]
Louisiana	1965	[4]
Mississippi	1990	[7]
**America (South)**		
Brazil	1988, 1991, 1994, 2017, 2019	[38,39,40,41,42,48]
Colombia	1994, 2001, 2010	[45,46,47]
Ecuador	1987, 2002, 2016	[13,43]
**Asia**		
Bangladesh	1971, 1978, 1979, 1983, 1990	[49,50,51,52]
China	2001, 2015, 2017, 2019, 2020, 2021	[18,19,20,21,22,53]
Indonesia	1993, 2015, 2018	[54,55,56]
India	1963, 1979, 1985, 1987, 1989, 1993, 1994 2000, 2004, 2005, 2006, 2007, 2010, 2011, 2017	[3,23,57,58,59,60,61,62,63,64,65,66,67,68,69]
Laos	1968	[70,71]
Malaysia	1994	[72]
Myanmar	1981, 2011	[73,74]
Nepal	2007, 2009	[16,35]
Pakistan	2003	[34]
Philippines	1994, 2001	[75,76]
Singapore	2001	[77]
Sri-Lanka	1997, 2001	[30,31]
Thailand	1971	[25]
Vietnam	1992, 1994	[33,78]
**Europe**		
Italy	2016, 2018	[2,14]

**Table 2:** Cultivated hosts of *Meloidogyne graminicola.*

Family	Species (Common Name)	Reference	Family	Species (Common Name)	Reference
Amaranthaceae	***Beta vulgaris*** (Beetroot)	[126]	Malvaceae	***Abelmoschus esculentus*** (Okra)	[124]
	***Spinacia oleracea*** (Spinach)	[12]	Musaceae	***Musa* sp.** (Banana)	[127]
Amaryllidaceae	***Allium cepa*** (Onion)	[76]		***M. acuminate*** (Dwarf banana)	[128]
	***A. tuberosum*** (Chive)	[129]	Poaceae	***Avena sativa*** (Oat)	[5]
	***A. fitsulosum*** (welsh onion)	[129]		***Hordeum vulgare*** (Barley)	[23]
Apiaceae	***Coriandrum sativum*** (Coriander)	[126]		***Oryza sativa*** (Rice)	[5,6]
Asteraceae	***Lactuca sativa*** (Lettuce)	[12]		***Saccharum officinarum*** (Sugarcane)	[12]
Brassicaceae	***Brassica oleracea*** (Cabbage)	[12]		***Sorghum bicolor*** (Sorghum)	[12]
	***B. oleracea* var. botrytis** (Cauliflower)	[128]		***Triticum aestivum*** (Wheat)	[10,123]
Cucurbitaceae	***Cucumis sativus*** (Cucumber)	[12]		***Zea mays***(Maize)	[12]
Fabaceae	***Glycine max*** (Soybean)	[122]	Solanaceae	***Capsicum frutescens*** (Chilli)	[130]
	***Phaseolus vulgaris*** (Common bean)	[5]		***C. annuum*** (Pepper)	[124]
	***Vigna adiate*** (Green gram)	[12]		***Solanum lycopersicum*** (Tomato)	[124]
	***V. unguiculata*** (Cowpea)	[12]		***S. melongena*** (Aubergine)	[124]

**Table 3:** Weeds hosts of Meloidogyne graminicola.

Family	Species (Common Name)	Reference	Family	Species (Common Name)	Reference
Alismataceae	***Alisma plantago*** (Common water- plantain)	[14]	Oxalidaceae	***Oxalis corniculata***	[128]
Amaranthaceae	***Alternanthera sessilis*** (Sessile joy weed)	[100]	Papillionaceae	***Melilotus alba*** (Yellow sweet clover)	[23]
	***Amaranthus spinosus*** (Spiny amaranth)	[40]	Plantaginaceae	***Scoparia dulcis*** (Licorice weed)	[122]
	***A. viridis*** (Slender amaranth)	[122]	Poaceae	***Agropyron repens*** (Quack grass)	[100]
Acanthaceae	***Rungia parviflora***	[128]		***Andropogon* sp.** (Beard grass)	[130]
Apiaceae	***Centella asiatica*** (Spade leaf)	[128]		***Alopecurus* sp.** (Foxtails)	[120]
Apocynaceae	***Catharanthus roseus*** (Periwinkle)	[12]		***A. carolinianus*** (Carolina foxtail)	[5]
Asteraceae	***Ageratum conyzoides*** (Billy-goat- weed)	[100]		***Brachiaria mutica*** (Buffalo grass)	[100]
	***Blumea* sp.**	[130]		***B. ramosa*** (Brown top millet)	[100]
	***Eclipta alba*** (False Daisy)	[130]		***Bothriochloa intermedia***	[100]
	***E. prostrata*** (Eclipta alba)	[131]		***Cynodon dactylon*** (Bermuda grass)	[126]
	***Grangea ceruanoides***	[130]		***Cymbopogon citratus*** (Lemon grass)	[128]
	***G. madraspatensis***	[130]		***Dactyloctenium aegyptiu***	[100]
	***Sphaeranthus* sp.**	[126]		***D. annulatum***	[23]
	***Sphaeranthus senegalensis***	[128]		***Digitaria filiformis*** (Crab grass)	[126]
	***Vernonia cinerea***	[128]		***D. longifolia*** (False couch grass)	[132]
Balsaminaceae	***Impatiens balsamina*** (Garden balsam)	[12]		***D. sanguinalis*** (Dewgrass)	[100]
Brassicaceae	***Brassica juncea*** (Brown mustard)	[12]		***Echinochloa colona***	[130]
	***Brassica* sp.**	[12]		***E. colonum***	[4]
Caryophyllaceae	***Spergula arvensis*** (Corn spurry)	[23]		***E. crus-galli*** (Barnyard grass)	[5]
	***Stellaria media*** (Chickweed)	[122]		***E. indica*** (Goose grass)	[130]
Commelinaceae	***Cyanotis cucullata*** (Roth)	[132]		***E. unioloides*** (Chinese love grass)	[132]
	***Commelina benghalensis***	[132]		***Eleusine coracana*** (Finger millet)	[126]
	***Murdannia keisak*** (Marsh dew flower)	[14]		***Eragrostis tenella***	[128]
Compositae	***Gnaphalium coarctatum***	[133]		***Imperata cylindrica*** (Spikegrass)	[128]
Cyperaceae	***Cyperus brevifolius*** (Kyllinga)	[126]		***Ischaemum rugosum*** (Saramolla)	[126]
	***C. compressus*** (Annual sedge)	[105]		***Leersia hexandra***	[134]
	***C. difformis*** (Variable Flatsedge)	[135]		***Oplismenus compositus***	[122]
	***C. imbricatus***	[126]		***Poa annua*** (Annual bluegrass)	[40]
	***C. odoratus***(Flats edge)	[136]		***Panicum dichotomiflorum***	[40]
	***C. pilosus*** (Fuzzy flats edge)	[128]		***P. miliaceum***	[122]
	***C. procerus***	[126]		***P. sumatrense***	[128]
	***C. pulcherrimus*** (Elegant s edge)	[126]		***P. repens***	[40]
	***C. rotundus*** (Purple nutsedge)	[100]		***Paspalum sanguinola***	[130]
	***Fimbristylis complanata***	[126]		***Paspalum scrobiculatum***	[126]
	***F. dichotoma***	[126]		***Pennisetum glaucum***	[128]
	***F. littoralis*** (Lesser fimbristylis)	[126]		***P. pedicellatum***	[128]
	***F. miliacea***	[130]		***P. typhoides*** (Pearl millet)	[122]
	***Fuirena ciliaris***	[126]		***Scirpus articulatus***	[126]
	***F. glomerata***	[126]		***Setaria italica*** (Foxtail millet)	[12]
	***Schoenoplectus articulatus***	[128]		***Sporobolus diander***	[100]
Euphorbiaceae	***Chamaesyce hirta*** (Asthma herb)	[136]	Polemoniaceae	***Phlox drummondii*** (phlox)	[12]
	***Phyllanthus urinaria***	[130]	Pontederiaceae	***Heteranthera reniformis***	[14]
Fabaceae	***Desmodium triflorum***	[122]		***Monochoria vaginalis***	[12]
	***Pisum sativum*** (Garden pea)	[12]	Portulacaceae	***Portulaca oleracea***	[122]
	***Trifolium repens*** (White clover)	[12]	Solanaceae	***Petunia* sp.**	[12]
	***Trigonella polyceratia***	[23]		***Physalis minima***	[100]
Hydrocharitaceae	***Hydrilla* sp.**	[132]		***Sida acuta*** (Broom grass)	[132]
Juncaceae	***Juncus microcephalus***	[137]		***Solanum nigrum***	[128]
Lamiaceae	***Leucas lavandulifolia***	[128]		***S. sisymbriifolium***	[128]
Linderniaceae	***Bonnaya brachiata***	[122,126]	Sphenocleaceae	***Sphenoclea zeylanica***	[126]
	***Lindernia* sp.**	[134]	Ranunculaceae	***Ranunculus* sp.** (Buttercup)	[105]
	***Vandellia* sp.**	[130]	Rubiaceae	***Borreira articularis***	[138]
Lythraceae	***Ammannia pentandra***	[126]		***Hedyotis diffusa***	[128]
Onagraceae	***Jussieua repens***	[130]			
	***Ludwigia adscendens*** (Primrose)	[134]			

## Data Availability

Data generated during this study are included in this article.
